# Genome wide exploration of the origin and evolution of amino acids

**DOI:** 10.1186/1471-2148-10-77

**Published:** 2010-03-15

**Authors:** Xiaoxia Liu, Jingxian Zhang, Feng Ni, Xu Dong, Bucong Han, Daxiong Han, Zhiliang Ji, Yufen Zhao

**Affiliations:** 1 The Key Laboratory for Chemical Biology of Fujian Province, Department of Chemistry, College of Chemistry and Chemical Engineering, Xiamen University, Xiamen 361005, Fujian, PR China; 2 School of Life Science, Xiamen University, Xiamen 361005, Fujian, PR China; 3 The Key Laboratory for Bioorganic Phosphorus Chemistry and Chemical Biology, Ministry of Education, Department of Chemistry, School of Life Sciences and Engineering, Tsinghua University, Beijing 100084, PR China

## Abstract

**Background:**

Even after years of exploration, the terrestrial origin of bio-molecules remains unsolved and controversial. Today, observation of amino acid composition in proteins has become an alternative way for a global understanding of the mystery encoded in whole genomes and seeking clues for the origin of amino acids.

**Results:**

In this study, we statistically monitored the frequencies of 20 alpha-amino acids in 549 taxa from three kingdoms of life: archaebacteria, eubacteria, and eukaryotes. We found that the amino acids evolved independently in these three kingdoms; but, conserved linkages were observed in two groups of amino acids, (A, G, H, L, P, Q, R, and W) and (F, I, K, N, S, and Y). Moreover, the amino acids encoded by GC-poor codons (F, Y, N, K, I, and M) were found to "lose" their usage in the development from single cell eukaryotic organisms like *S. cerevisiae *to *H. sapiens*, while the amino acids encoded by GC-rich codons (P, A, G, and W) were found to gain usage. These findings further support the co-evolution hypothesis of amino acids and genetic codes.

**Conclusion:**

We proposed a new chronological order of the appearance of amino acids (L, A, V/E/G, S, I, K, T, R/D, P, N, F, Q, Y, M, H, W, C). Two conserved evolutionary paths of amino acids were also suggested: A→G→R→P and K→Y.

## Background

The origin of life arising from either proteins or nucleic acids has been argued for nearly half century. Putting the "Chicken or Egg" question aside, there exist some unsolved problems. Which amino acid(s) appeared first in the prebiotic environment? What cause the different usage of amino acids in modern organisms? To address these questions, a number of hypotheses and theories, e.g. mutation drifts and natural selection, have been proposed. Multiple factors, such as genetic codes, physicochemical properties, mutation-selection equilibrium, amino acid biosynthesis, etc, are likely related to the variation of amino acid usage in organisms [1,2]. Since there is no way to trace geological evidence in the way scientists normally use in chronicling the evolution of organisms, an alternative path is needed to seek a clue from current living organisms.

Observation of amino acid composition in proteins was recently applied as a statistical approach in facilitating various investigations of the evolution of genetic codes [3], the origin of amino acids [1,2,4-6], the co-evolution of amino acids and genetic codes [7], the evolution of protein families [8-10], the conservation of subcellular location [11], the prediction of protein secondary structure [12-14], the natural selection of protein charge [15], the correlation between gene expression level and protein function [16], the kinship of different taxa [17], the molecular mechanisms of dinosaur extinction [18], the lifestyles of organisms [19], and even the tracing of the Latest Universal Ancestor (LUA) of life [4-6]. Recently, some research groups have successfully applied genomic information on monitoring amino acid composition linked with various biological phenomena [1,5,11,17,20]. It is beyond question that an insight into the evolution of amino acids on a genomic scale can extend our knowledge about molecular evolution and the origin of life. In this study, 549 genomes from three kingdoms of life were adopted to investigate statistically the patterns of amino acid usage during evolution. Also, clues for the origin of amino acids in prebiotic environment and their co-evolution with genetic codes were explored.

## Results and Discussion

### Chronological order of appearance of amino acids

Which amino acid(s) appeared first in the prebiotic environment? To address this question, we might go back to the first life form in the world. When the first simple life was formed, most amino acid biosynthesis processes had not become fully functional. The environment was the only source to acquire amino acids and other fundamental bio-molecules for life. As a consequence, the amino acid composition of the early life was mainly determined by the amino acid content in the "prebiotic soup" with no or little bias on selection of amino acids. It was assumed that the "early" amino acids had higher concentration in the primitive environment than that of "late" amino acids, thus had higher composition in early life form. Retrospectively, if the amino acid composition of the early life form was estimated, it could be used to determine the amino acid concentration in the environment and further deduced the chronological order of amino acid appearance.

**Figure 1:**
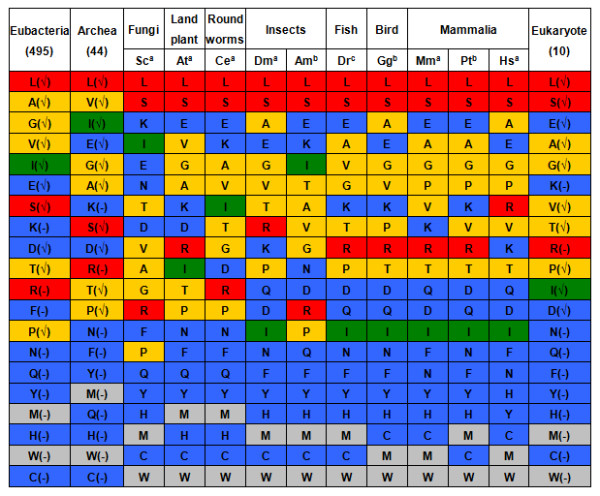
**Rankings of amino acid composition in three kingdoms of life**. Frequency rankings for 20 alpha amino acids in eubacteria, archaebacteria and 10 selected eukaryotic representatives: *Saccharomyces cerevisiae *(Sc), *Abrabidopsis thaliana *(At), *Caenorhaditis elegans *(Ce), *Drosophila melanogaster *(Dm), *Apis mellifera *(Am), *Danio rerio *(Dr), *Gullus gallus *(Gg), *Mus musculus *(Mm), *Pan troglodytes *(Pt), and *Homo sapiens *(Hs). The status of genome was labeled as: ^(a) ^complete annotation, ^(b) ^draft assembly, ^(c) ^in progress. The numbers of genomes from the three life kingdoms of archaebacteria, eubacteria and eukaryotes are presented after the kingdom names. For each taxa or life kingdom, the amino acids were ranked in descending order of their frequencies in the whole proteome. Amino acids are colored by the number of codons: red, orange, green, blue, and gray stands for 6, 4, 3, 2, and 1 codons respectively. An amino acid is marked with "√ " if it was detected by Miller's spark tube experiments, otherwise, marked with "-".

According to this assumption, we estimated the amino acid composition of early life form by genome-wide monitoring of amino acid usage in modern organisms. As observation, amino acid compositions are substantially varied not only inter-species between three kingdoms of life but also inter-species within a kingdom (Additional Files 1). This caused difficulty in deducing a consensus amino acid composition for the LUA. In an additional construction of taxa kinship hierarchy based on amino acid composition in the three kingdoms of life (data not presented), we found that taxa from same life kingdom tended to gather together. Therefore, an ancestral amino acid usage was determined separately by kingdoms as follows: Amino acids were first scored from 20 to 1 in terms of descending order of their frequencies in each designated species. The sum of the scores for each amino acid was then calculated and ranked by kingdom of life (Figure 1). Integrating the data of three life domains, a generally-agreed rank of amino acid frequency was achieved. This rank was considered as the estimated amino acid composition of the early life form. Accordingly, a possible chronological order of amino acid appearance was thus proposed in descending order: L, A, V/E/G, S, I, K, T, R/D, P, N, F, Q, Y, M, H, W, C. This order agrees well with the previous findings of Miller's experiments [21] that ten "early" amino acids (A, D, E, G, I, L, P, S, T, V) rank in the top 12. It is slightly different from Trifonov's study (G/A, V/D, P, S, E/L, T, R, N, K, Q, I, C, H, F, M, Y, W) which was determined by comparison of forty different single-factor criteria and multi-factor hypotheses [22]. The assignment of "early or late" amino acids was also supported by additional correlation analyses between physiochemical properties of amino acids and their genetic codes (Additional File 2). Both this study (Figure 1 & Additional File 2) and previous evidence [21,22] supported the assignment of aromatic amino acids (F, W, and Y) as "late" amino acids. Moreover, the effects of a high pH primitive environment on genetic codes in early earth environment determined that there were more early basic amino acids than early acidic amino acids [15].

### Co-evolution of amino acids and genetic codes

It has been suggested earlier that amino acid composition was determined largely by existing genetic codes [23]. In our study, the relationship between amino acids and codons has also been studied. As shown in Figure 1, the amino acids with more codons are "favored" by proteins. This phenomenon was observed not only in eukaryotes, but also in most representatives of eubacteria and archaebacteria. Two six-codon owners, leucine and serine, are the most frequently-used amino acids in all selective eukaryotic species. Arginine is also a six-codon amino acid, but its frequency of use is much lower than expected (averagely ranking 9^th ^in eukaryotes, 10^th ^in archaebacteria, and 11^th ^in eubacteria). The under-utilization of arginine is as yet mechanistically unclear, but it may be related to its physiochemical properties and roles in protein functions. All the four-codon amino acids (A, G, V, T, and P) are positioned in the middle zone, and most of the two-codon amino acids and all the one-codon amino acids are used less often.

**Figure 2:**
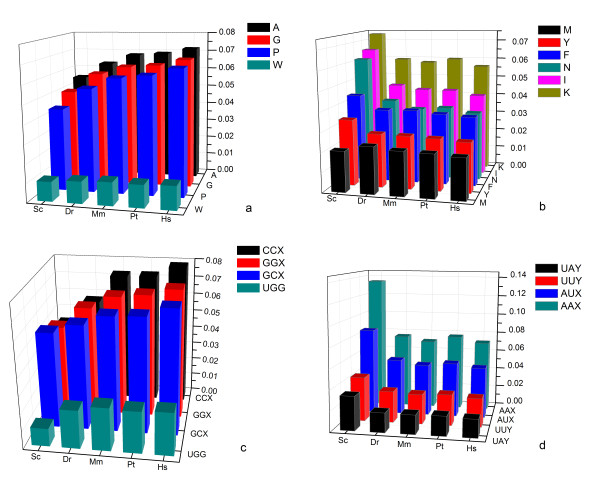
**Trends in usage of GC-rich/poor codons and their encoded amino acids**. Trends in usage of amino acids encoded by GC-rich codons (2a), amino acids encoded by GC-poor codons (2b), GC-rich codons (2c), and GC-poor codons (2d) over five eukaryotic organisms, *Saccharomyces cerevisiae *(Sc), *Danio rerio *(Dr), *Mus musculus *(Mm), *Pan troglodytes *(Pt), and *Homo sapiens *(Hs). Amino acids encoded by GC-rich codons (P, A, G, W) increase their usage from lower organisms to higher organisms while amino acids encoded by GC-poor codons (F, Y, N, K, I, M) in general decrease. All GC-rich codons (CCX, GCX, GGX, and UGG) increase their usage over the five eukaryotic organisms, while GC-poor codons (AAX, AUX, UUY and UAY) decrease their usage over eukaryotic species.

Previous research has proposed that all amino acids with declining frequencies were the first to be incorporated into the genetic code [1]. To examine this finding, 3D charts of amino acid frequency-codon relationship were prepared (Figure 2), including 5 selected eukaryotic representatives in the same branch of Darwin Evolution (*S. cerevisiae*, *D. rerio, M. musculus, P. troglodytes*, and *H. sapiens*). Interestingly, all the amino acids encoded by GC-rich codons (definitively, CCX/GCX/GGX/UGG [24,25]), i.e. (P, A, G, and W), increased their frequencies from *S. cerevisiae *to *H. sapiens*; while all the amino acids encoded by GC-poor codons (definitively, AAX/AUX/UAX/UUX), i.e. (F, Y, N, K, I, M), decreased. These results conflict with the previous findings of Jordan and his team [1] that P, A, G, and E 'lose' in protein evolution. The disagreement may be caused by different protein data sets adopted by these two studies and different evolutionary history of amino acids in three kingdoms of life (Figure 1 & Additional file 1). Further statistics of codons showed that the numbers of GC-rich codons (CCX/GCX/GGX/UGG) increased from *S. cerevisiae *to *H. sapiens*, while the GC-poor codons (AAX/AUX/UAY/UUY) decreased (Figure 2c, 2d & Additional file 3). Similar results have also been obtained in previous studies using different approaches [26,27].

**Figure 3:**
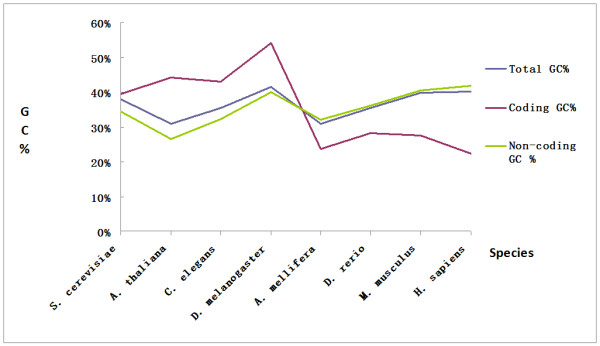
**Comparison of GC% in coding regions and non-coding regions**. The GC% of both coding regions and non-coding regions in the whole genomes of eight organisms (*S. cerevisiae*, *A. thaliana*, *C. elegans*, *D. melanogaster*, *A. mellifera*, *D. rerio*, *M. musculus*, and *H. sapiens*) were compared. The GC% in the coding regions is higher than that of the non-coding regions in lower organisms, while reversed in higher organisms.

Additionally, we calculated the correlation coefficients between "random" amino acid frequencies following from a uniform usage of codons of the universal genetic code and amino acid compositions of the modern organisms (Additional File 1). As in previous findings [17], all eukaryotic representatives showed a higher correlation coefficient, indicating the small selection of amino acid composition of proteins in eukaryotes. However in eubacteria and in archaea, correlation coefficients varied from 0.05 to 0.9, suggesting that some microbials show a significant selection of amino acids for their proteins. The substantial variety of selection pressure in microbials may be explained by factors such as particular living environments, frequent mutation, rapid generation, etc. To have an overview of how GC content could affect amino acid usage, we compared the GC% of both coding regions and non-coding regions in the whole genomes of eight organisms. Statistically, the coding regions in lower eukaryotes have rather higher net GC content than the non-coding regions, but this is manifestly reversed in higher organisms (*A. mellifera*, *D. rerio*, *M. musculus*, and *H. sapiens*), where it can be seen that the net GC content of the coding regions decreases from lower eukaryotes to higher eukaryotes (Figure 3 & Additional file 4). But our previous finding (Figure 2c) indicates that the usage of GC-rich codons increases from *S. cerevisiae *to *H. sapiens*. So the decrease in G and C content in coding region in higher eukaryotic species might come from the decrease in the usage of intermediate-GC codons (defined in ref 24). All these suggest the GC rich condons are favorable in proteins even under the pressure of the decrease in GC content.

**Figure 4:**
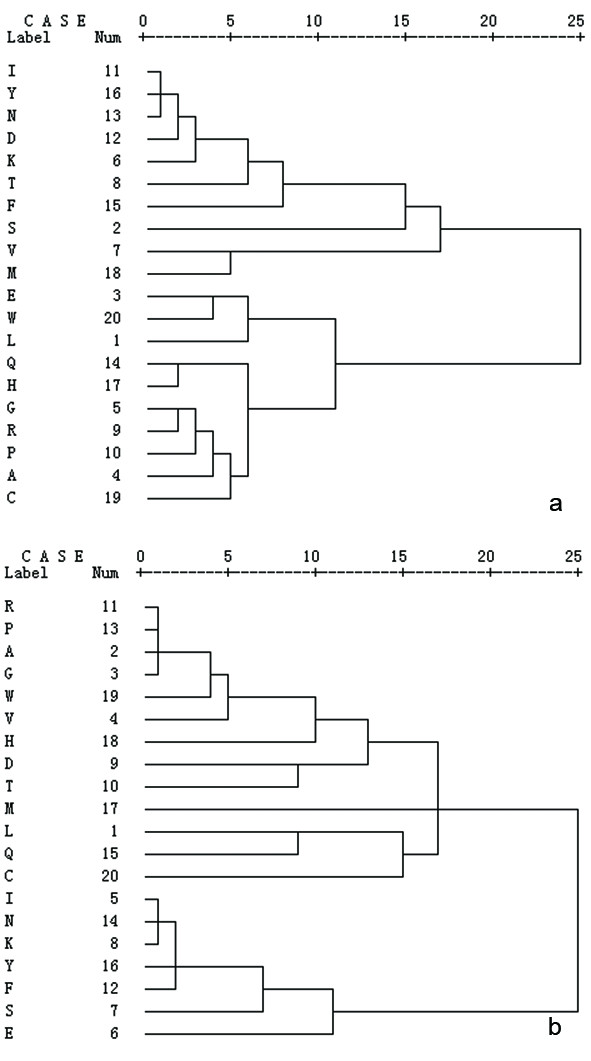
**Kinship clusters of amino acids**. Kinships of amino acids based on their frequencies over eukaryotes (4a) and eubacteria (4b). Amino acids were clustered using SPSS11.0 software by calculating the Pearson correlation coefficient of their frequencies over 10 eukaryotic organisms or 495 eubacteria. As illustrated in 4a and 4b, the 20 alpha-amino acids were divided into two clusters: cluster 1 (A, C, G, H, P, Q and R) and cluster 2 (D, I, K, N, and Y) for eukaryotes and cluster 1 (A, G, P, R, V, and W) and cluster 2 (F, K, I, N, and Y) for eubacteria. The number besides the amino acids indicates their frequency order in Figure 1.

To seek a connection between the frequency changes of amino acids and genetic codes, correlation analyses were established for eukaryotes and eubacteria (Figure 4). It was shown that all amino acids encoded by GC-rich codons (P, A, G, W) clustered together both in the eukaryote and eubacteria. All the amino acids encoded by GC-poor codons (F, Y, N, K, I, M) in bacteria gathered into a cluster except methionine. These results further support the co-evolution of amino acids and genetic codes.

### Kinship of amino acids

**Figure 5:**
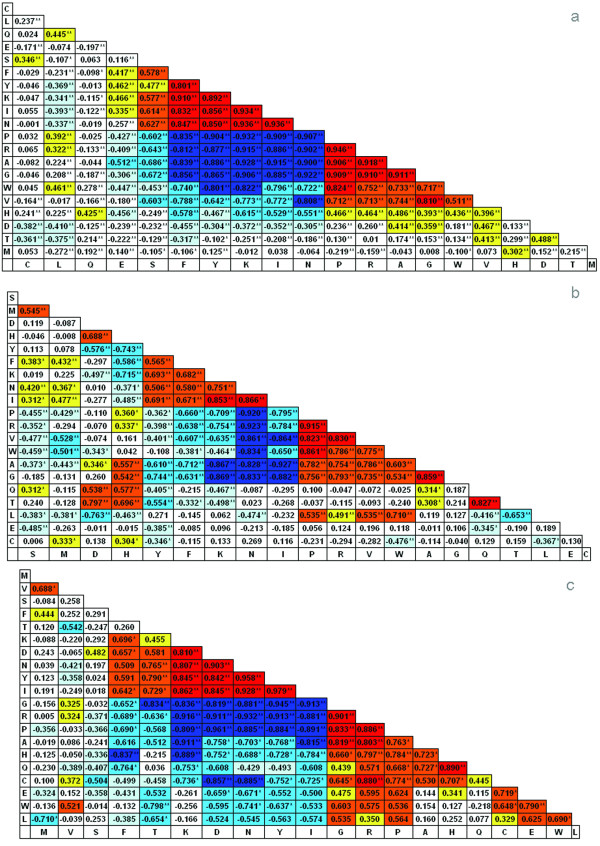
**Triangle tables of correlation coefficients between amino acids**. Triangle tables of correlation coefficients between amino acids in kingdoms of eubacteria (5a), archaebacteria (5b), and eukaryotes (5c). The correlations between 20 amino acids were determined by calculating Pearson correlation coefficients (*r*) of amino acid frequencies over 495 eubacteria, 44 archaebacteria, and 10 eukaryotic representatives respectively. Each correlation between a pair of amino acids was colored: red (*r *> 0.8), orange (0.5 <*r *< 0.8), yellow (0.3 <*r *< 0.5), blue (*r *< -0.8), light blue (-0.8 <*r *< -0.5), light green (-0.5 <*r *< -0.3) and white for others. The significance (2-tailed) was also listed in the table: ** indicates that the correlation is significant at the 0.05 level (2-tailed), and * indicates that the correlation is significant at the 0.01 level (2-tailed).

**Figure 6:**
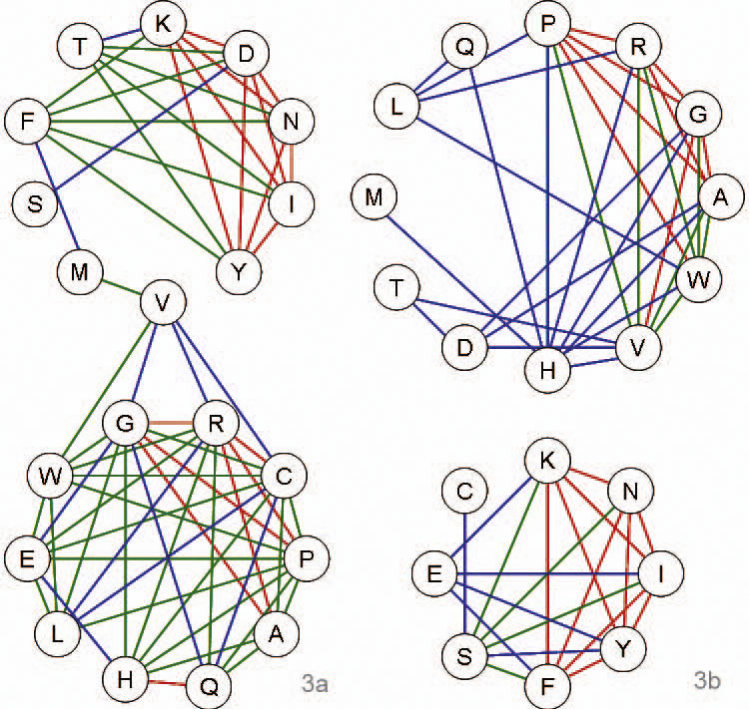
**Correlated clusters of amino acids**. Clusters determined by correlation analyses of amino acid composition in eukaryotes (6a) and eubacteria (6b). Amino acids with correlated frequencies are connected by lines and colored according to Pearson correlation coefficients (*r*): red (*r *> 0.8), green (0.5 <*r *< 0.8), and blue (0.3 <*r *< 0.5). It is noted that 20 alpha-amino acids group into two clusters: cluster 1 (D, F, I, K, M, N, S, T, V and Y) and cluster 2 (A, C, E, G, H, L, P, Q, R and W) for eukaryotes (6a) and cluster 1 (A, D, G, H, L, M, P, Q, R, T, V and W) and cluster 2 (C, E, F, I, K, N, S and Y) for eubacteria (6b). Amino acids in the same cluster are suggested to have common evolutionary history.

It is challenging to describe how amino acids develop from "early" to "late". A plausible approach is to seek hints from the correlation of amino acid composition. This is based on the assumption that two amino acids are evolutionarily connected if they are correlated in frequencies across species. In this study, Pearson Correlation Coefficients (*r*) of amino acid compositions were calculated separately within three kingdoms of life, and the results were arranged and presented in triangular tables (Figure 5). It was observed that amino acids were gathered into several zones: two zones of (A, G, P, R) and (K, I, N, Y) in eubacteria (Figure 5a), two zones of (P, R, V) and (N, I, K) in archaebacteria (Figure 5b), and two zones of (D, K, I, N, Y) and (A, G, P, R) in eukaryotes (Figure 5c). Within a zone, the amino acid pairs show highly positive or negative correlation (colored in red or deep blue, respectively) in frequency. The correlations was further analyzed and illustrated in kinship maps (Figure 6a and Figure 6b). As illustration, 20 alpha-amino acids were assigned into two distinct clusters: (cluster 1 (D, F, I, K, M, N, S, T, V and Y) and cluster 2 (A, C, E, G, H, L, P, Q, R and W)) for eukaryotes and cluster 1 (A, D, G, H, L, M, P, Q, R, T, V and W) and cluster 2 (C, E, F, I, K, N, S and Y) for eubacteria. The amino acids are connected by lines of different correlations. It is evident that the positively correlated amino acids normally locate in the same cluster, which suggests a common evolutionary history or functional connection. In contrast, those negatively correlated amino acids were separated into different clusters. It indicates a distinct evolutionary history or functional competition. As the evolution of amino acids may have proceeded independently in the three kingdoms of life, it is understandable that amino acids show different kinships in eukaryote and eubacteria. However, conserved linkages were observed that some amino acids are always gathered together: (A, G, H, L, P, Q, R, W) and (F, I, K, N, S, Y). This suggests that amino acids may have evolved mainly in two separate paths.

**Figure 7:**
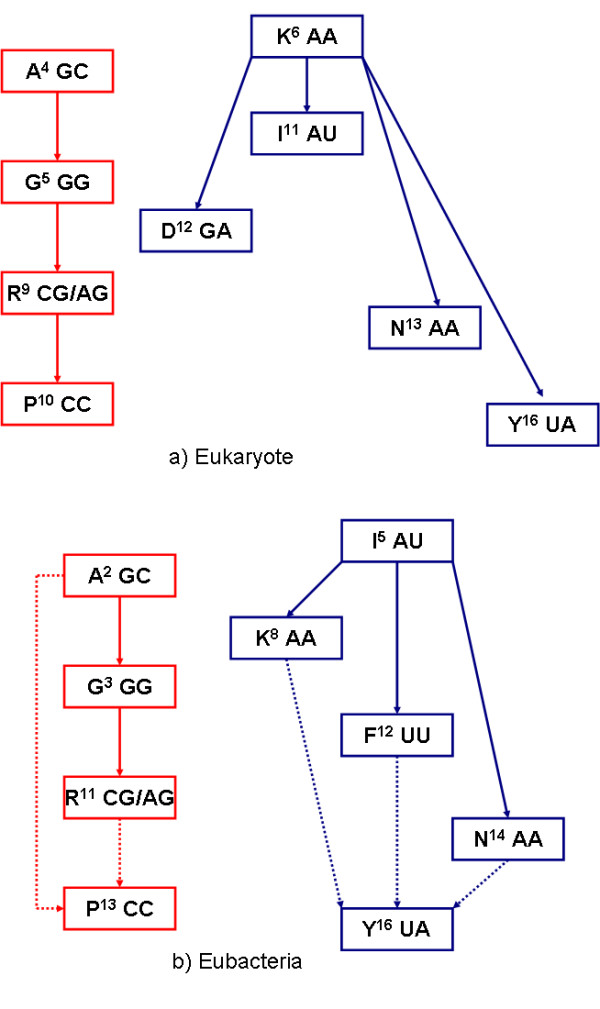
**Putative evolutionary paths of amino acids**. Putative evolutionary paths of amino acids based on data of eukaryotes (7a) and eubacteria (7b). Each block contains an amino acid, its ranking of frequency (the number beside the amino acid), and first two characters of its codons. The continuous arrow indicates the direction of evolution and the dotted arrow indicates more than one possible direction of evolution. It seems that new amino acids tend to derive from "parent" amino acids by one-base codon change. It is noted that evolutionary paths of amino acids in eukaryotes (7a) and eubacteria (7b) are not exactly the same, however, conserved evolutionary paths are observed: A→G→R→P and K→Y.

It was also found (Figure 2) that amino acids with similar codons are inclined towards having similar usage during evolution, e.g. P/R, and N/I/Y. These amino acids may have a common evolutionary origin. Accordingly, the potential evolutional paths of amino acids were drawn using the following criteria. Firstly, amino acids with a Pearson correlation coefficient above 0.8 were designated as lineal consanguinity, either paternity or fraternity. Secondly, the assignment of kinship should agree with the chronological order of amino acids. Thirdly, the transition between amino acids is favored by one-codon mutation, especially the last codon in the codon triplet. These results are illustrated in Figure 7. It can be seen that the evolutionary paths of amino acids in eukaryotes and eubacteria are not always coincident. However, two independent and conserved evolutional paths were found: A→G→R→P and K→Y.

## Conclusion

Our study agrees with previous research that statistical analysis of amino acid composition in proteins is a feasible route to global understanding of the physiological function of living organisms and the mystery encoded in whole genomes. However, proper evaluation of "real" amino acid usage in a modern taxa may be affected by a series of factors, including, time scale of evolution, frequency of organism generation, diverse living environments, chronological order of amino acid appearance, bias of genetic codes, gene mutation frequency, mutation-selection equilibrium, preference of physico-chemical properties, difficulty of biosynthesis, co-evolution of amino acids and genetic codes, incomplete annotation of genomes, existence of "retired" genes and pseudogenes in genomes, and other as yet unrecognized reasons. Many of these factors are currently unpredictable and incalculable and thus have been ignored in this study. It can be concluded that statistical observation of amino acid composition in modern proteomes is an alternative means for broadening our current knowledge on the origin of life.

## Methods

### Data

Whole genome information of 549 prokaryotes (including 495 eubacteria and 44 archaebacteria) and 10 eukaryotic representatives (*Saccharomyces cerevisiae, Abrabidopsis thaliana, Caenorhaditis elegans, Drosophila melanogaster, Apis mellifera, Danio rerio, Gullus gallus, Mus musculus, Pan troglodytes, and Homo sapiens*) were derived from NCBI genome resource. Taxonomy of these selected organisms, their unique NCBI entry IDs and annotation versions were listed in the Additional File 1.

## Methods

The composition of amino acids in each genome was measured by calculating the frequencies of amino acids against all open reading frames (ORFs) in the whole genome. The frequency of each amino acid was determined by(1)

where N_*i *_is the number of amino acid *i *in genome ORFs, and N_*total *_is the sum of all 20 amino acids in genome ORFs. This calculation is subjected to the assumption that the ORF assignments in selected genomes are correct. The Pearson correlation coefficients (*r*) of amino acids frequency data from the three kingdoms was computed by Bivariate Correlations procedure of software SPSS 13.0, at significance level 0.01 and 0.05.(2)

Where X and Y are the two random amino acids; ρ_X, Y _is the correlation coefficient between X and Y; μ_X _and μ_Y _are expected values; σ_X _and σ_Y _are standard deviations; E is the expected value operator; cov means covariance.

## Authors' contributions

ZJ is the designer and supervisor of this project. YZ is the co-supervisor of this project and helped to revise the manuscript. XL is the major researcher and prepared the manuscript. JZ, FN, XD, BH, DH were involved in data collection and analyses at different stages of this project. All authors have read and approved the final manuscript.

## Supplementary Material

Additional file 1 **Table S1**. Frequencies of 20 alpha-amino acids in 495 eubacteria, 44 archaebacteria and 10 eukaryotic representatives. The archaebacteria were prefixed with "*". NCBI entry IDs and annotation versions were provided with taxa names. The correlation coefficients between "random" amino acid frequencies following from uniform codon usage and amino acid compositions of the modern organisms were also given. Click here for file

Additional file 2 **Table S2**. Average frequencies of genetic codes for basic or acidic amino acids in three kingdoms of life. Average frequencies of genetic codes for basic or acidic amino acids in kingdoms of archaebacteria, eubacteria, and eukaryotes respectively. The basic amino acids are Histidine (H), Lysine (K), and Arginine (R), and the acidic amino acids are Aspartic acid (D) and Glutamic acid (E). AFGC stands for Average frequency of genetic codes. Click here for file

Additional file 3 **Table S3**. Frequencies of 64 genetic codes in 495 eubacteria, 44 archaebacteria and 10 eukaryote representatives. Click here for file

Additional file 4 **Table S4**. The GC% of both coding regions and non-coding regions in the whole genomes of eight organisms (*S. cerevisiae, A. thaliana, C. elegans, D. melanogaster, A. mellifera, D. rerio, M. musculus, and H. sapiens*). Click here for file
